# 加速肺康复外科，需要精准治疗吗？

**DOI:** 10.3779/j.issn.1009-3419.2017.08.08

**Published:** 2017-08-20

**Authors:** 国卫 车, 伦旭 刘

**Affiliations:** 610041 成都，四川大学华西医院胸外科 Department of Thoracic Surgery, West China Hospital, Sichuan University, Chengdu 610041, China

**Keywords:** 加速康复外科, 加速肺康复外科, 精准医学, Enhanced recovery after surgery, Enhanced lung recovery after surgery, Precision medcine

## Abstract

加速康复外科（enhanced recovery after surgery, ERAS）理念已得到医护的认可，多学科协作是ERAS实践的前提。但现有临床方案实施效果却差异很大，原因何在呢？分析主要原因是统一方案不一定适应于所有手术患者，是否存在“过度医疗”呢？换言之，ERAS是否也需要精准治疗呢？本文主要以肺手术后的加速肺康复（enhanced lung recovery after surgery, ELRAS）为例，分析ERAS精准治疗的必要性及达到的临床效果。一是术前需要肺康复训练的患者人群界定要准确（高危因素的评估标准要精准），肺康复训练为核心，以降低术后并发症为目的；二是术前有明确症状的患者，术前肺康复训练方案也应精准，以控制症状和改善患者生活质量为目的；三是术前无症状及严重相关伴随疾病患者，以优化围手术期流程（精准去掉不必要的操作）为主，以提高患者住院舒适度和缩短平均住院日为目的。总之，加速肺康复外科不是做“加法”而是做“减法”。

## Abstract (en)

The concept of enhanced recovery after surgery (ERAS) has already been accepted by almost all the clinicians and nurses, the practice of which is based on interdisciplinary cooperation. The reason is still unclear why the effect of ERAS varies a lot though the same ERAS scheme is used. The main cause may be the same ERAS scheme can not be suitable for different patients. In other words, does ERAS also need to conform to Precision Medicine Theory? This study is focused on the necessity and clinical efficacy of "Precision ERAS" performed in lung cancer patients. The conclusions are the following: first of all, an accurate judgment of patients who need ERAS should be done properly before surgery, which means that the high risks assessment should be done accurately. Secondly, a specific ERAS scheme should be carried out in each independent patient who has obvious clinical symptoms in order to alleviate clinical symptoms and improve the ptients' quality of life (QOL). Thirdly, for the asymptomatic patitents who also don't have severe concomitant diseases, process-optimized ERAS should be selected to make patients feel more comfortable and shorten the average length of stay (ALOS). To summary, "subtraction" instead of "addition" should be considered when performing ERAS.

加速康复外科（enhanced recovery after surgery, ERAS）主要通过降低手术创伤和减少机体应激反应，近期达到缩短住院时间和降低医疗费用，并实现“no pain and no risk”的终极目的^[1, 2]^。国内外已发布多种术后ERAS指南或专家共识，如胃切除手术，肝胆胰手术等^[3]^，但临床应用的依从性和效果却差异很大^[4]^？分析其主要原因可能有以下几方面，一是ERAS方案大同小异，应用人群“精准性”和“个体性”差；多学科协作临床应用方案时，对部分相对简单患者是否存在“过度治疗”值得思考？二是ERAS方案评价标准在临床应用过程中简单化（多数医生及医院主要以降低并发症和缩短住院日为标准），对合并高危因素患者，不能简单以住院时间作为评价是否加速康复的标准。三是现有的ERAS方案过分强调了围手术期医疗过程（如术前宣教、目标性导向性液体治疗）及症状管理（疼痛等），而忽略了术前高危因素评估及术前准备（如术前康复训练及相关疾病的治疗）。四是术后评价标准ERAS的标准也缺乏精准性，如常用的心肺并发症，目前心肺并发症无统一标准，且多沿用内科评价标准（如术后肺炎），不能准确体现手术自身带来的问题，还是真正的术后并发症^[5]^。这些问题一方面导致ERAS临床效果难以准确评价，另外也降低了ERAS方案的依从性。

以肺外科手术为例，微创技术和精准切除、损伤控制和流程优化的现代外科理念为ERAS的施行奠定了理论和实践基础^[4, 5]^，但患者术前均存在不同种类和程度的伴随疾病，术中麻醉、单肺和肺挫裂伤等使术后并发症发生率高，因此需要对不同患者实施“个体化”的ERAS方案来指导临床实践^[5]^。因此，本文从以下3个方面分析ERAS精准治疗的必要性及需要达到的临床效果。一是术前合并高危因素的患者，需要术前肺康复训练，以降低术后并发症为目的；二是术前有症状的患者，需要术前控制症状及肺康复训练，以控制症状和改善患者生活质量为目的；三是术前无症状及严重相关伴随疾病患者，以优化围手术期流程为主，以提高患者住院舒适度和缩短平均住院日为目的。同时探讨加速肺康复外科（enhanced lung recovery after surgery, ELRAS）可能实现的途径，旨在为实现肺外科手术ERAS的规范化、标准化提供参考意见。

## 1 高危因素评价标准的精准性

围手术期患者合并高危因素^[6]^，主要包括两类，术前主要是患者自身因素（如年龄等），生活习惯（如吸烟等），并存疾病（如高血压等）；术中主要是因手术需要而导致的危险因素，如麻醉时间过长（包括手术时间长），手术创伤（术中肺挫裂伤、失血过多等）；术后处理相关症状治疗时，因药物或治疗手段导致，如激素应用时出现消化道溃疡等。本节主要集中在术前及术中高危因素如何精准定义，术后症状处理相关危险因素放在症状管理论述。

### 1.1 患者自身危险因素^[7-13]^

自身危险因素主要源于患者身体状况、生活习惯及共存疾病。

#### 1.1.1 年龄

年龄作为危险因素，标准差异大；影响因素如生理与实际年龄、性别、共存疾病等。年龄≥75岁作为高危因素标准（若患者无吸烟史或吸烟指数 < 200年支且戒烟时间 > 8周）。

#### 1.1.2 吸烟史

以下三项满足一项：①吸烟指数≥800年支且戒烟时间 > 2周，不考虑年龄因素；②吸烟指数≥200年且戒烟时间 > 15天支，同时年龄≥60岁；③吸烟指数≥400年且戒烟时间 > 15天支，同时年龄≥45岁。

#### 1.1.3 气管定植菌（airway bronchial colonization）

以下三项满足一项：①年龄≥75岁；②吸烟指数≥800年支；③重度慢性阻塞性肺疾病（chronic obstructive pulmonary disease, COPD）。

#### 1.1.4 气道高反应性（airway high response, AHR）

以下四项满足一项：①支气管舒张试验阳性；②心肺运动试验（cardiopulmonary exercise test, CPET）过程中出现干啰音或哮喘或SpO_2_下降大于15%；③服用抗过敏药物或激素；④爬楼梯训练前后PEF值下降大于15%。

#### 1.1.5 呼气峰值流量（peak expiratory flow, PEF）

PEF < 250 L/min。

#### 1.1.6 肺功能临界状态或低肺功能^[5]^

肺功能临界状态是指：①FEV_1_ < 1.0 L；②美国外科肿瘤学学会（American College of Surgeons Oncology Group, ACOSOG）Z4099/RTOG标准: FEV_1_%：50%-60%或年龄 > 75岁和一氧化碳弥散量（carbon monoxide diffusing capacity, DLco）50%-60%；③美国胸内科医生学会（American College of Chest Physicians, ACCP）标准：预计术后FEV_1_ < 40%或DLco < 40%。

#### 1.1.7 肥胖

身体质量指数（body mass index, BMI）指数≥28。

#### 1.1.8 肺部基础疾病及其他胸部疾病

术前合并结核等其他病变引起的肺间质纤维化等^[6]^。

#### 1.1.9 既往治疗史

如术前接受过放疗和（或）化疗，或长期应用激素，以及既往有胸部手术史及外伤史等。

#### 1.1.10 健康状况和其他危险因素

各种原因引起的营养不良、贫血等，代谢性疾病如糖尿病，其他器官如心、肝、肾等功能不全亦是手术后气道炎症及肺部并发症的危险因素。

### 1.2 医疗相关危险因素^[7, 9, 11, 12]^

医疗危险因素主要指麻醉、手术过程中的危险因素。

#### 1.2.1 手术时间长

以下两项满足一项：①麻醉时间（从气管插管成功到气管插管拔除）≥4 h；②手术时间（从切皮开始到切口缝合完成）≥3 h。

#### 1.2.2 肺挫裂伤重

术后PaO_2_/FiO_2_的比值< 40 kpa（300 mmHg）。

## 2 肺康复训练方案的精准性

肺康复训练方案的精准性体现在两方面：一是肺康复训练方法；二是肺康复训练时间。

### 2.1 肺康复训练方案^[14-21]^

#### 2.1.1 药物康复

##### 2.1.1.1 抗生素应用

致病性气管定植菌的存在，术前需要应用敏感抗生素治疗（国内以G-），需要有明确证据（痰培养结果）；但临床取得明确细菌学证据困难，研究发现血清SP-D（serum surfactant protein D）浓度与致病性气管定植菌存在具有相关性^[20, 21]^。目前抗生素应用仍需按卫计委规定标准执行。

##### 2.1.1.2 祛痰药

口服或雾化吸入均可，但均需按照袪痰药的说明书应用。

##### 2.1.1.3 消炎药或平喘药

消炎药主要是指雾化吸入用激素类药；平喘药是指雾化吸入用支气管扩张剂等或口服类。

#### 2.1.2 物理康复

##### 2.1.2.1 激励式肺量计吸气训练（必需）

在非睡眠时间，每2 h重复一组训练，每组进行6次-10次训练，然后休息。

##### 2.1.2.2 功率自行车运动训练

运动量控制在呼吸困难指数（Borg）评分5分-7分之间，每次约15 min-20 min，每天2次。若没有条件可选择登楼梯训练。

##### 2.1.2.3 登楼梯训练（可选）

每天2次，每次15 min-30 min，疗程3 d-7 d。功率自行车运动训练和登楼梯训练二者选一。

### 2.2 肺康复训练时间^[14, 15, 17, 18]^

肺康复训练时间以3 d、7 d、14 d作为参考。以PEF作为评价指标，以PEF较训练前增加10%作为训练时间的标准。

## 3 围手术期流程管理的精准性

### 3.1 麻醉精准性^[22-24]^

精准化麻醉如何在临床上应用呢？主要体现在以下几方面：①根据病种进行“个体化”的麻醉，如非插管全麻胸腔镜下交感神经烧灼术治疗多汗症或气胸等。②根据手术方式选择麻醉方法，胸腔镜肺叶切除术（video-assisted thoracic surgery, VATS）手术时间短，有时可选择非插管、单腔管等。③气管插管拔管时机也应“个体化”，手术顺利且时间短的患者最好术后立即拔除气管插管，部分患者可在复苏室拔除，个别需要呼吸机支持的患者才需要到重症监护室拔除。

### 3.2 管道应用的精准性

#### 3.2.1 尿管应用^[25-28]^

胸部手术患者，若有术后尿潴留的高危因素之一，均需要应用尿管：①年龄（不分性别）≥75岁；②前列腺重度增生；③尿道手术史；④腹部手术史；⑤手术时间 > 4 h。若无以上高危因素，则术中不需要留置尿管。同时若根据术前评定标准，不置尿管则术前宣教和术中及术后处理均有相应措施。

#### 3.2.2 胸腔引流管^[29-32]^

胸腔引流管的精准应用体现在以下几方面；①单管引流取代双管引流（只有脓胸或术中肺漏气严重才考虑用双引流管）。②应用细引流管，有研究表明16 F引流管的引流效果等同于28 F-32 F不影响切口愈合。③术后引流管的拔除尽早，而是若无漏气，300 mL/d也可拔除。④也有报道术后不应用引流管的报道，但是需要术后排气，多数只是术后确认无气体漏出后，马上拔掉，但是需要选择病例且严密监测。

## 4 术后症状管理的精准性

肺癌术后症状评估量表临床应用可信度高且效度可靠，临床研究发现胸腔镜肺癌肺叶切除术后常见症状有疼痛、咳嗽及气短。

### 4.1.1 疼痛管理^[4, 5, 30]^

疼痛已被作为患者第5生命体征，应该给予更多关注，但目前似乎对症止痛处理过多。为追求“无痛”而过分应用镇痛方法，尤其是要多模式镇痛也要用“减法”。精准镇痛应体现在以下几方面：①针对疼痛原因进行处理，如胸腔引流管导致的疼痛，最好早期拔掉引流管^[21]^镇痛不充分或过度。②麻醉师或疼痛专业医生对病人进行评估，立足于围手术期疼痛管理，不但有效镇疼，且降低因疼痛导致的并发症。如围手术期合理应用甾体类止痛药同样可以达到吗啡类药的效果，且显著降低恶心、呕吐反应。③疼痛评估体系与方法主观性强，导致用药合理性差，缺乏围手术期统筹安排。④止痛药应用的合理优化，个体化^[33, 34]^。

### 4.2 咳嗽管理^[35, 36]^

咳嗽是肺术后常见的症状，严重影响患者术后的生活质量。目前缺乏术后咳嗽的客观评价标准和治疗策略，我们借助于呼吸内科评价急性和慢性咳嗽的中文版莱斯特咳嗽量表（Mandarin Chinese version of the Leicester Cough Questionnaire, LCQ-MC）初步研究表明：LCQ-MC可以用作肺术后咳嗽程度的评估，尤其是符合中国文化背景及肺癌术后发生咳嗽的原因和特点修订的人肺癌术后LCQ-MC咳嗽生命质量问卷（简化版），更加符合临床应用和评价。根据LCQ-MC咳嗽生命质量问卷（简化版）对肺术后患者咳嗽的客观评价，发现其影响因素和围手术期及术后干预措施，是咳嗽精准评价和治疗的前提。主要体现在以下几方面：①LCQ-MC咳嗽生命质量问卷（简化版）临床应用中，使量表不断优化且操作性更强。②针对肺癌术后咳嗽患者的影响因素，应用不同的围手术期预防措施。如胸腔镜肺癌肺叶切除术后咳嗽的主要影响因素有麻醉时间和术前患者有咳嗽，术前有咳嗽患者需要术前进行干预（肺康复训练）；手术时间长的患者，一方面术中尽量缩短麻醉及手术时间，另一方面术后积极采取预防咳嗽的治疗方案（如雾化吸入或口服相关药物等）。③根据肺术后患者咳嗽的特点，发现有效的控制与治疗的药物，达到区别于内科的针对外科病因的有效的精准治疗。

### 4.3 气短管理^[33]^

肺癌患者手术出院后气短（气喘、动则气促和轻度呼吸困难）是影响日常生活的主要原因。有效和精准治疗方案必需建立在客观评价的基础之上，如术后气短主观评分：评价患者对气短的主观感受；术后步行距离：评价患者运动后气短耐受程度；术后肺功能：评价患者通气功能，确定患者气短严重程度。根据不同病因，一方面调整围手术期采取预防及治疗措施；另一方面术后应用不同的肺康复方案进行有效治疗。目前这方面的研究不多。

## 5 ERAS临床标准评价的精准性^[22, 23, 37, 38]^

ERAS方案临床应用成功与否，不能仅用是否降低并发症及缩短平均日作为标准，而应更加精准，才有助于临床上准确评价ERAS方案适用性。本文建议如下：高危因素患者，以术前肺康复训练为核心（家庭、社区、胸外科、康复科或呼吸科），以术后相关并发症（严重并发症）是否降低作为评价标准；术前有症状患者，以控制症状为核心（对症处理、康复训练及中药治疗等），术后以控制症状和改善生活质量为评价标准；正常患者（体检患者，术前无明显症状者），以优化围手术期流程为核心（如麻醉、管道管理等），以住院舒适度和缩短平均住院日作为评价标准。

## 6 问题与方向

ERAS的理念已被广泛接受，且临床效果已初步显现，这种新理念正在改变外科实践，尽管很慢，但却是外科学发展的方向。目前存在的问题有：①医患的依从性差，一方面是却乏可临床应用的成熟方案，另一方面也是习惯和观念更新较慢。②过分强调减少术中和术后患者身体对外科手术的严重应激反应，而对术前风险评估和干预，优化治疗共存病症重视不够。③缺乏合理、客观、统一的评价ERAS方案成功与否的标准体系。这些问题也是我们的研究方向，需要多学科及多中心的大量研究，利用大数据不断优化ERAS方案，使ERAS方案的可操作、可评估和可重复性更强。
